# A genome-wide association study in catfish reveals the presence of functional hubs of related genes within QTLs for columnaris disease resistance

**DOI:** 10.1186/s12864-015-1409-4

**Published:** 2015-03-17

**Authors:** Xin Geng, Jin Sha, Shikai Liu, Lisui Bao, Jiaren Zhang, Ruijia Wang, Jun Yao, Chao Li, Jianbin Feng, Fanyue Sun, Luyang Sun, Chen Jiang, Yu Zhang, Ailu Chen, Rex Dunham, Degui Zhi, Zhanjiang Liu

**Affiliations:** Fish Molecular Genetics and Biotechnology Laboratory, Aquatic Genomics Unit, School of Fisheries, Aquaculture and Aquatic Sciences and Program of Cell and Molecular Biosciences, Auburn University, Auburn, AL 36849 USA; Department of Biostatistics, University of Alabama at Birmingham, Birmingham, AL 35294 USA

**Keywords:** Columnaris, Disease resistance, Bacteria, Immunity, GWAS, PI3K, Hybrid, Fish, QTL

## Abstract

**Background:**

Columnaris causes severe mortalities among many different wild and cultured freshwater fish species, but understanding of host resistance is lacking. Catfish, the primary aquaculture species in the United States, serves as a great model for the analysis of host resistance against columnaris disease. Channel catfish in general is highly resistant to the disease while blue catfish is highly susceptible. F2 generation of hybrids can be produced where phenotypes and genotypes are segregating, providing a useful system for QTL analysis. To identify genes associated with columnaris resistance, we performed a genome-wide association study (GWAS) using the catfish 250 K SNP array with 340 backcross progenies derived from crossing female channel catfish (*Ictalurus punctatus*) with male F1 hybrid catfish (female channel catfish *I. punctatus* × male blue catfish *I. furcatus*).

**Results:**

A genomic region on linkage group 7 was found to be significantly associated with columnaris resistance. Within this region, five have known functions in immunity, including *pik3r3b, cyld-like, adcyap1r1, adcyap1r1-like*, and *mast2*. In addition, 3 additional suggestively associated QTL regions were identified on linkage groups 7, 12, and 14. The resistant genotypes on the QTLs of linkage groups 7 and 12 were found to be homozygous with both alleles being derived from channel catfish. The paralogs of the candidate genes in the suggestively associated QTL of linkage group 12 were found on the QTLs of linkage group 7. Many candidate genes on the four associated regions are involved in PI3K pathway that is known to be required by many bacteria for efficient entry into the host.

**Conclusion:**

The GWAS revealed four QTLs associated with columnaris resistance in catfish. Strikingly, the candidate genes may be arranged as functional hubs; the candidate genes within the associated QTLs on linkage groups 7 and 12 are not only co-localized, but also functionally related, with many of them being involved in the PI3K signal transduction pathway, suggesting its importance for columnaris resistance.

## Background

Commercial production of catfish (*Ictalurus spp.*) accounts for approximately 60% of US aquaculture production (www.ers.usda.gov). However, due to devastating diseases, the catfish industry has been hindered by unprecedented challenges. Diseases can cause losses of up to one third of the industry each year. *Flavobacterium columnare*, a Gram-negative bacterium, is the causative agent of columnaris disease, which is very common in wild and cultured freshwater fish worldwide [1]. This pathogen can infect a variety of fish species through mucosal attachment points on the gill and skin, causing external erosion and necrosis [2]. The bacterium can also enter the blood stream and invade the internal organs [3]. The economically important foodfish channel catfish (*Ictalurus punctatus*) and the other members of the family Ictaluridae are extremely susceptible to columnaris disease. Columnaris disease is considered as one of the most important diseases in the catfish industry, causing tens of millions of dollars in losses every year [2]. Therefore, improving disease resistance is a major goal for genetic stock enhancement using various approaches including strain selection, crossbreeding, hybridization, and transgenics [4].

Heterosis is an important genetic force that contributes to world food production. Interspecific hybrids are particularly effective in generating significant heterosis [5]. Hybrids of catfish have been investigated for about 50 years [6]. Among all possible combinations, only one cross (female channel catfish × male blue catfish) exhibited better performance than its parental species in growth rate and feed conversion efficiency [7].

Various techniques have been used to dissect the genes responsible for production and performance traits in aquaculture species. For instance, traditional QTL mapping was conducted to locate the region associated with resistance to infectious pancreatic necrosis virus in Atlantic salmon (*Salmo salar*) [8-13]. Utilizing RNA-seq, Li et al. [14] characterized the role of catfish intestinal epithelial barrier following enteric septicemia of catfish (ESC, *Edwarsiella ictaluri*) challenge. Wang et al. [15] conducted bulked segregant analysis to study candidate gene locations and allele-specific expression associated with ESC resistance in catfish. However, genetic analysis of resistance against bacterial diseases such as columnaris has been limited.

In the last decade, various studies were conducted aiming at elucidating the mechanisms of columnaris entry, immune evasion, and the host response to the disease. Some studies have been conducted on the modes and dynamics of columnaris adhesion [16,17]. In recent years, the application of RNA-seq has allowed a significantly greater level of understanding of the complexities of columnaris induced gene expression [18]. Several central signatures following infection were revealed by gene expression enrichment analysis and gene pathway analysis of differentially expressed genes [18]. For instance, Beck et al. [19] revealed that rhamnose-binding lectin was induced dramatically after infection, which was correlated with columnaris susceptibility. Peatman et al. [20] carried out RNA-seq analysis to compare basal and post-challenge differences in expression between susceptible and resistant channel catfish lines. Some genes involved in critical innate immunity, such as iNOS2b, lysozyme C, IL-8, and TNF-alpha were constitutively expressed higher in resistant than in susceptible catfish gill tissues. In contrast, secreted mucin forms, rhamnose-binding lectin, and some mucosal immune factors were found to be expressed at higher levels in the susceptible catfish line than in the resistant line. Despite these efforts, the knowledge of molecular mechanism of columnaris resistance is still limited.

Genome-wide association mapping allows detection of markers closely linked to QTLs. Although QTL mapping is well-suited for family-based samples, association studies, especially genome-wide association studies, can potentially offer higher mapping resolution using markers with higher density. Moreover, recent developments in GWAS methodologies have offered mature software packages for association analysis. Although GWAS has been widely utilized in economically important plants [21] and animals [22,23], it has been seldom applied in fish. To identify the QTLs in the hybrid catfish related to columnaris resistance, a genome-wide association study using backcross hybrid catfish was conducted, and here we report the identified QTLs and their associated genes within the highly associated genomic regions.

## Results

### Mortality rate

**Figure 1:**
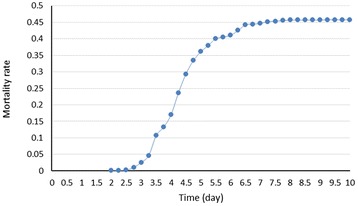
**Mortality rate of hybrid catfish after** ***Flavobacterium columnare*** **infection.**

The accumulative mortality rate was 45.7%. A total of 1,200 channel catfish from a mix of six families were pooled and communally challenged. The mortalities started 46 hours after challenge and peaked approximately 218 hours after challenge (Figure 1). Based on the selective genotyping method [24], the blood samples of the first 169 dead fish were collected, serving as the “susceptible” fish. After 12 days of challenge, 652 fish survived. From the survivors, 171 fish without symptoms of columnaris were randomly collected as the “resistant” fish.

### Sample structure

**Figure 2:**
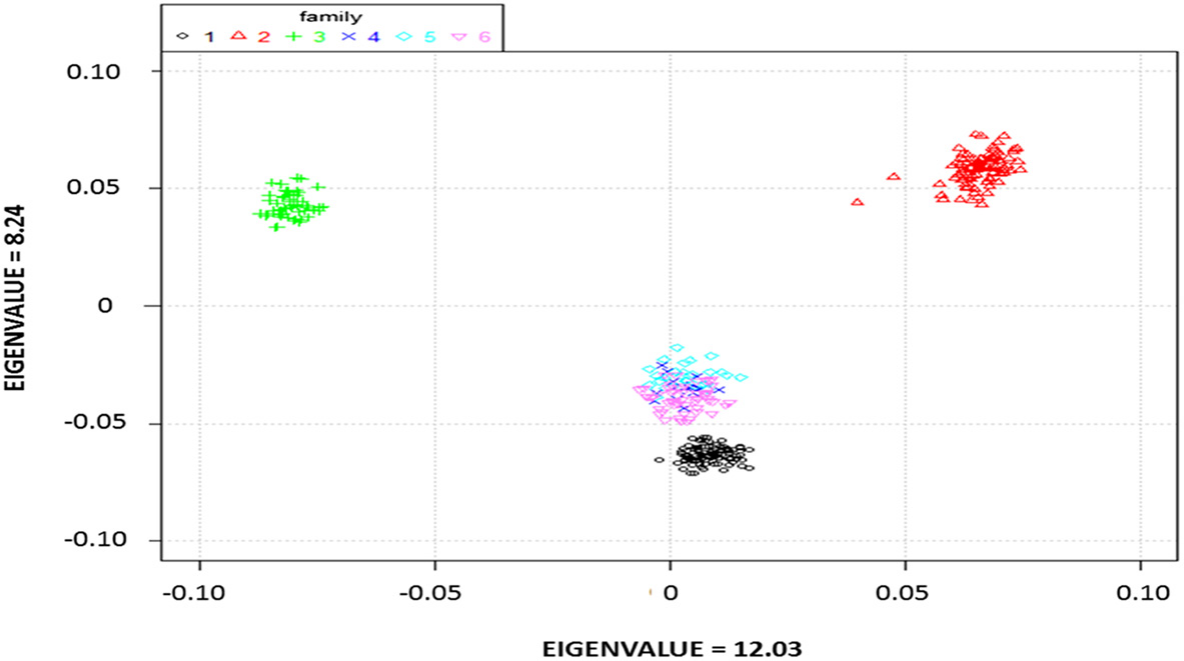
**Sample structure identified by PCA with the first two principal components.** The coordinates are the first two principal components.

The founders of the 6 families were known before the experiment, but the offspring were mixed for communal culture. In order to assign the genotyped fish to each of the six families, cluster analysis was conducted according to the IBS kinship matrix (Table 1). With known family pedigree, principal component analysis was conducted using eigenvalues as coordinates to visualize the sample structure. As shown in Figure 2, apparently, families 4, 5, and 6 were highly related, while families 1, 2, and 3 were distantly related.

**Table 1:** **The pedigree information of catfish samples used in this study**

**Family ID**	**Dam**	**Sire**	**Sample number**	**Susceptible sample number**	**Resistant sample number**
1	Channel 1	Hybrid 1	96	48	48
2	Channel 2	Hybrid 1	95	47	48
3	Channel 3	Hybrid 2	55	28	27
4	Channel 4	Hybrid 1	14	8	6
5	Channel 5	Hybrid 1	27	18	9
6	Channel 6	Hybrid 1	53	20	33

### Analysis of linkage disequilibrium (LD) blocks

The LD block was defined as a set of contiguous SNPs with the minimum pairwise r^2^ value exceeding 0.50 [22]. The number of independent SNP markers and LD blocks was 14,420. Thus the threshold P-value for genome-wide significance was 0.05/14420=3.47e-6 (−log_10_ (P-value)=5.46). The threshold P-value for the significance of “suggestive association”, which allows one false positive effect in a genome-wide test, was 1/14420=6.93e-5 (−log_10_ (P-value)=4.16).

### Linkage groups with associated QTLs for columnaris resistance

**Figure 3:**
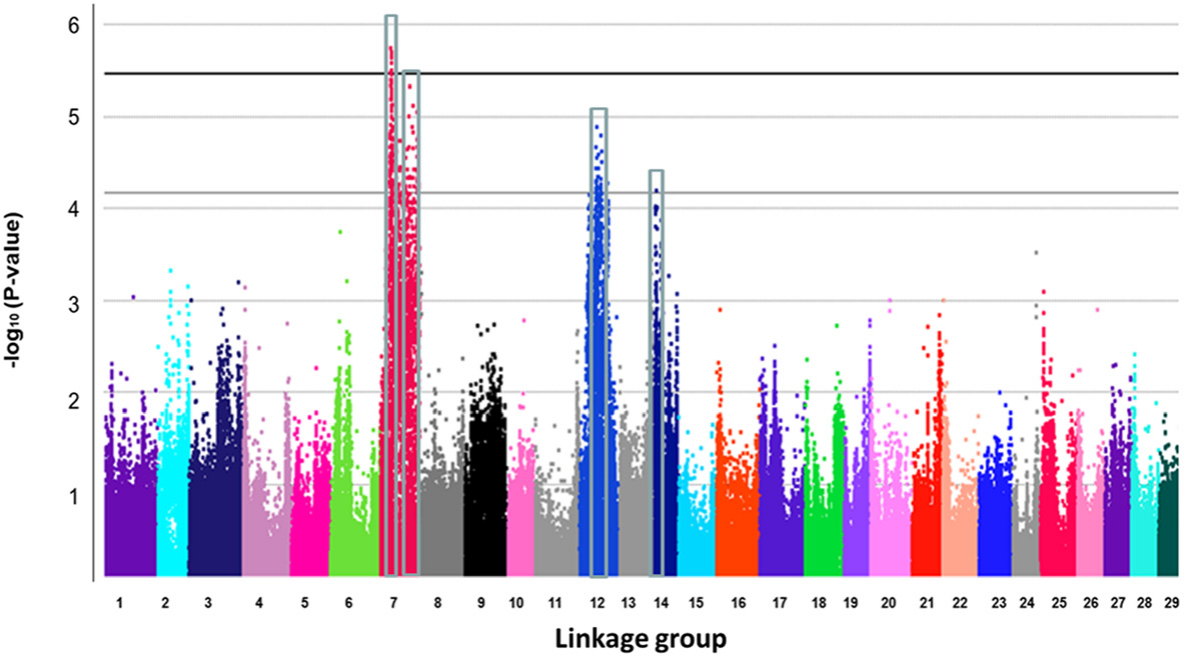
**Manhattan plot of genome-wide association analysis for columnaris disease resistance.** The black solid line indicates the threshold P-value for genome-wide significance. The gray solid line indicates the threshold P-value for the significance of “suggestive association”. The four boxes indicate the associated regions.

A Manhattan plot constructed using the marker positions and the corresponding -log_10_ (P-value) is shown in Figure 3. Linkage group 7 harbors markers that are statistically significant at the genome level (−log_10_ (P-value) > 5.46). A second genomic region on linkage group 7 appears to harbor suggestively associated markers, but is not statistically significant at the genome level. In addition to linkage group 7, linkage groups 12 and 14 appear to harbor SNP markers that are also suggestively associated with columnaris resistance, although not statistically significant at the genome level (Figure 3).

### Genomic region with significantly associated QTL for columnaris resistance

**Figure 4:**
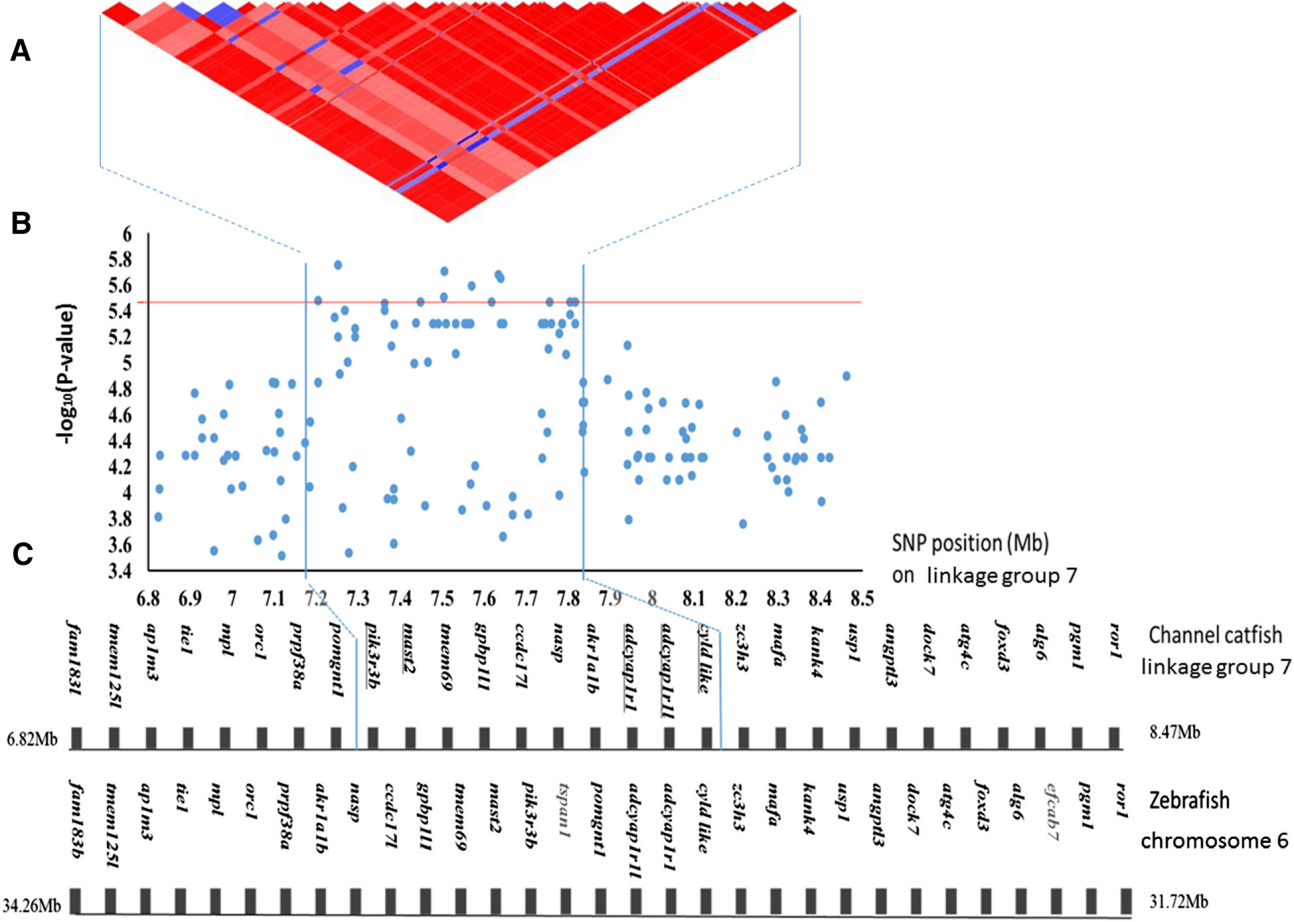
**Regional genome scan for the QTL significantly associated with columnaris resistance on linkage group 7. A)** Heat map of the LD between the most significant SNPs in the QTL region. **B)** Regional–log10 (P-value) plot for the QTL**.** The horizontal red line indicates the threshold P-value for genome-wide significance. **C)** Synteny analysis comparing catfish and zebrafish. Candidate gene names are underlined. Genes with gray names are located in the region of zebrafish but not channel catfish.

Additional analysis was conducted with the chromosomal region where the significantly associated QTL is located on linkage group 7. A set of 12 most significant SNPs are listed in Table 2. These SNPs are all significantly associated with columnaris resistance at the genome level (−log_10_ (P-value) > 5.46). These SNPs are all located in a genomic region on linkage group 7 from 7,203,819 bp to 7,817,023 bp, spanning a total of approximately 620 Kb. Their minor allele frequencies vary between 0.24-0.39, and their nearby genes are listed in Table 2. Because family-based population with 8 founders were utilized, the haplotypes extend very long regions as expected (Figure 4).

**Table 2:** **The significantly associated SNPs on linkage group 7**

**SNP ID**	**Position (bp)**	**-Log10 (P-value)**	**Nearest gene**
AX-86060479	7252290	5.75	upstream of *pik3r3b*
AX-85347098	7505396	5.70	*mast2* intron
AX-86056344	7633758	5.68	*adcyap1r1* exon
AX-85337705	7639440	5.65	*adcyap1r1* exon
AX-85377681	7569451	5.59	downstream of *akr1a1b*
AX-85265763	7503336	5.51	*mast2* intron
AX-85240370	7203819	5.48	upstream of *pik3r3b*
AX-85319066	7447662	5.47	*mast2* intron
AX-85346312	7618017	5.47	*adcyap1r1* intron
AX-85378689	7755734	5.47	*zc3h3* intron
AX-85233717	7803806	5.47	*zc3h3* intron
AX-85260766	7817023	5.47	*zc3h3* intron

EMMAX was used to investigate the contribution of the significantly associated QTL to the phenotype. Because of high correlation between SNPs in one locus (Figure 4 and Section Correlation of the SNPs associated with columnaris resistance), when analyzing the fraction of variance explained by the QTL, we chose only the most significant SNP (AX-86060479) to represent this region, which could explain 6.6% of the phenotypic variance. Nevertheless, binary phenotype and selective genotyping used in our study may cause potential overestimation of the QTL effect.

Based on the SNPs placed on the catfish 250 K SNP panel [25], the parental origins of the SNPs could be determined. All the 12 significant SNPs are interspecific, which means on these loci, two species are simply fixed for alternate alleles. The resistant genotypes for the 12 SNP loci are homozygous with both alleles being originated from channel catfish.

### Genes located within the significantly associated QTL region for columnaris resistance

The genes within the genomic region harboring the significant SNPs associated with columnaris resistance were annotated by BLAST analysis against the non-redundant protein database [26]. To provide additional supporting evidence for the proper annotation of the region, syntenic analyses were also conducted to compare the gene contents in the genomic neighborhood around the significant SNPs. As shown in Figure 4, the conserved synteny was identified between the catfish and zebrafish genomes. The flanking genes of catfish are all conserved with zebrafish except *efcab7* and *tspan1* are missing in catfish and the gene orders are slightly different.

Within the 620 Kb region containing the most significant SNPs, a total of 10 genes were identified (Figure 4). Of the 10 genes, five genes were found to have known functions in immunity, and these include phosphatidylinositol 3-kinase regulatory subunit gamma b (*pik3r3b*)*,* cylindromatosis*-*like (*cyld-like),* pituitary adenylate cyclase-activating polypeptide type 1 receptor (*adcyap1r1), adcyap1r1-like*, and microtubule-associated serine and threonine kinase 2 (*mast2*). In order to be sure all the candidate genes were included in the analysis, the extended genomic region was examined, and no gene was found with known function in immunity.

### Suggestively associated QTLs

In addition to the significantly associated QTL on linkage group 7, three additional genomic regions were identified to contain SNPs suggestively associated with columnaris resistance (−log_10_ (P-value) > 4.16), although not statistically significant (Figure 3). As shown in Table 3, SNPs with relatively low P values were identified from the three suggestively associated regions on linkage groups 7, 12 and 14.

**Table 3:** **The most significant SNPs with the closest and candidate genes in 3 suggestively associated regions**

**Linkage group**	**SNP ID**	**SNP position (bp)**	**-log**_**10**_ **(P-value)**	**Gene name**	**Gene position (bp)**
7	AX-85432363	19715765	5.01	cysteinyl leukotriene receptor 2	19913410, 19914493
	AX-85417541	20399460	5.33	guanine nucleotide-binding protein (G protein) subunit beta 1*	19923349, 19927379
	AX-85231041	22082916	4.89	voltage-dependent calcium channel subunit alpha 2/delta 2^#^	20083452, 20300502
	AX-85406722	22298185	4.83	hyaluronidase 2	20409454, 20413226
	AX-85205344	22410724	5.12	tumor suppressor candidate 2	20415941, 20421024
	AX-85278425	25230627	5.12	diphosphoinositol polyphosphate phosphohydrolase 1	20432914, 20443526
				protein kinase C and casein kinase substrate in neurons protein 1	20469564, 20487008
				N-terminal EF-hand calcium-binding protein 1	22274849, 22324281
				alpha-1-syntrophin	22377042, 22408256
				probable G-protein coupled receptor 27	25065312, 25066872
12	AX-85394454	12067569	4.89	phosphatidylinositol 3-kinase regulatory subunit 5^†^	12246367, 12272207
	AX-85211547	14746472	4.79	phosphatidylinositol 3-kinase regulatory subunit 6^†^	12281638, 12300055
				guanine nucleotide-binding protein subunit beta 3*	12763269, 12767890
				voltage-dependent calcium channel gamma 6 subunit^#^	14286927, 14292214
				chondroitin sulfate proteoglycan 4	13668415, 13668975
14	AX-85234783	1601158	4.20	Spectrin beta chain, non-erythrocytic 2	1614033, 1619504

The paralogs are marked by different symbols following the gene names.

The first suggestively associated region is on linkage group 7. About 12 Mb downstream of the significantly associated region on linkage group 7, there is another locus suggestively associated with columnaris resistance. A series of SNPs on that region exhibit relatively low P values with the lowest ones listed on Table 3, with –log_10_ (P-value) ranging from 4.83-5.12. In addition to linkage group 7, there are SNPs on linkage group 12 that reach suggestive genome-wide significance (P-value < 6.93e-5). The most significant SNP could explain 5.5% of the variance. Compared with the region strongly associated with the columnaris resistance on linkage group 7, the region detected on linkage group 12 is larger. According to the linkage map [27], the recombinant frequency is very low on this region of linkage group 12. The distance of two most significant SNPs is 2.68 Mb on linkage group 12 expanding about 2 centimorgans, while in catfish, on average, 1 cM is equivalent to approximately 250 Kb. According to the suggestively associated interspecific SNPs on linkage group 12, the resistant genotypes of the SNPs are homozygous with two channel catfish alleles, like the SNPs on linkage group 7. On linkage group 14, there is only one SNP reaching suggestive Bonferroni genome-wide significance, explaining 4.6% of the variance. The candidate genes surrounding the most significant SNPs of these 3 loci are listed in Table 3.

### Correlation of the SNPs associated with columnaris resistance

Conditioned analyses were conducted to examine the correlation of the SNPs associated with columnaris resistance [23]. Genotypes of the most significant SNP (AX-86060479) on linkage group 7 were included as a covariate in the mixed linear model. After conditioning, the –log_10_ (P-value) of all the other SNPs on linkage group 7 dropped below 2.0, while the SNP P-values remained generally unchanged on the other linkage groups. On the linkage groups 12 and 14, there were also strong correlations among these associated SNPs within the same linkage group (data not shown).

## Discussion

In this study, for the first time, we identified a significantly associated QTL on linkage group 7 and three additional suggestive QTLs on linkage groups 7, 12, and 14 for columnaris resistance. The significant QTL on linkage group 7 was narrowed down to a small region of 620 Kb. Therefore, in spite of being just an initial quantitative analysis for the complex disease resistance trait, this work is very significant because it has set the foundation for fine mapping of the columnaris resistance genes, providing basis for application of genome technologies for catfish aquaculture through marker- or genome-based selection. Additional fine mapping using larger or more families could narrow down the candidate genes underlining columnaris disease resistance.

A number of genes involved in the PI3K pathway were found to be within the significantly associated genomic region of 620 Kb, suggesting the involvement of PI3K pathway in the resistance against columnaris. Among the 10 genes found in the 620 Kb region, five were involved in PI3K gene pathway. These are *pik3r3b, cyld-like, adcyap1r1, adcyap1r1-like*, and *mast2*. The phosphatidylinositol 3-kinase regulatory subunit gamma b (*pik3r3b*) is the closest gene to the most significantly associated SNP (AX-86060479) (Table 2). Although the causative SNP could be within any one of the 10 genes found in the 620 Kb region, the fact that PI3K pathway was reported to be involved in immunity and resistance makes them particularly interesting as candidate genes [28]. In addition, many genes found in the suggestive QTL regions were also involved in the PI3K pathway (see below), further increasing the likelihood of PI3K pathway involvement in the resistance against columnaris.

PI3 kinases have been known to play important roles in innate and adaptive immunity [28-31]. For example, PI3K activity is important for NF-κB pathway activation in different mechanisms [32,33]. It was also shown that the infectious agents can manipulate the PI3K pathway to create a favorable environment by various mechanisms [34]. PI3K is required for modifying the cytoskeleton dynamics, regulating membrane traffic, coordinating exocytic membrane insertion and pseudopod extension, which could be utilized by some pathogenic bacteria for entry into host cell [34-38]. Ireton et al*.* [34] showed that the protein InlB from *Listeria monocytogenes* is an agonist of mammalian PI3K, which causes rapid increases in cellular amounts of PI (3,4) P_2_ and PI (3,4,5) P_3_. Lambotin et al. [38] found *Neisseria meningitidis* requires cortactin recruitment by triggering a PI3K/Rac1 signaling to elicit an efficient invasion in non-phagocytic cells. Kierbel et al. [39] reported *Pseudomonas aeruginosa* requires the PI3K and Akt pathway for internalization. It was reported that *Porphyromonas gingivalis* could activate PI3K, which blocks phagocytosis and promotes inflammation [40]. In addition, it was shown that blockade or deficiency of PI3Kγ significantly enhanced resistance against *Leishmania mexicana*, which revealed the unique role for Class I_B_ PI3K in parasite invasion [41]. The fact that *pik3r3b* is closest to the most significant SNP makes it an interesting candidate for future studies.

**Figure 5:**
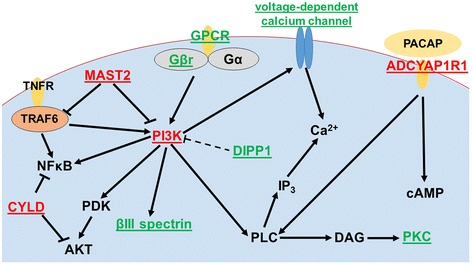
**Signal transduction pathways involving PI3Ks and the other candidate genes.** The candidate genes in the significant QTL are in red. The candidate genes in the suggestive QTLs are in green.

In addition to *pik3r3b* gene, four other genes (*cyld-like, adcyap1r1, adcyap1r1-like*, and *mast2*) in the PI3K pathway (Figure 5) found within the 620 Kb region containing the significant QTL have also been shown to play important roles in immunity. CYLD is a deubiquitylating enzyme that negatively regulates various signaling pathways by cleavaging lysine 63-linked polyubiquitin chains from several specific substrates [42]. For example, CYLD could regulate inflammation and the innate immune response via its inhibition of NF-κB activation [43]. Besides that, CYLD is a deubiquitinating enzyme for Akt and suppressed Akt activation [44]. Gao et al. [45] reported that CYLD also plays a role in the regulation of microtubule dynamics.

Pituitary adenylate cyclase-activating polypeptide type I receptor (ADCYAP1R1) is the pituitary adenylate cyclase-activating polypeptide (PACAP) specific receptor [46]. PACAP could activate adenylate cyclase and phospholipase C (PLC) through an interaction with ADCYAP1R1 and stimulates cAMP and inositol phosphate formation [47,48]. In fish larvae, Lugo et al. [49] reported that the PACAP influences immune functions. They observed an elevated level of nitric oxide synthase-derived metabolites and total immunoglobulin M concentration in serum of juvenile catfish and tilapia after intraperitoneal injection of PACAP.

Microtubule-associated serine and threonine kinase 2 (MAST2) can interact with phosphatase and tensin homolog deleted on linkage group 10 (PTEN) which antagonizes PI3K-dependent signaling pathways [50,51]. MAST2 inhibits TNF receptor associated factor 6 (TRAF6) activity [52], which represents a molecular bridge for innate immunity and adaptive immunity [53]. For example, TRAF6 could activate PI3K-dependent cytoskeletal changes and activate IκB kinase (IKK) in response to proinflammatory cytokines [54]. Taken together, PI3 kinases themselves, or genes involved in PI3K pathway could play important roles in disease resistance.

Interestingly, many genes mapped within the suggestively associated QTL regions on linkage groups 7, 12, and 14 are functionally related to the genes mapped within the significant QTL region on linkage group 7, further supporting the possibility that PI3K pathway may be important for disease resistance against columnaris (Table 3, Figure 5). On linkage group 7, cysteinyl leukotriene receptor 2 is a G protein-coupled receptor (GPCR) with various functions such as modulation of chemokine gene transcription and calcium signaling [55,56]. G beta gamma activates the class I_B_ p110 gamma/p101 PI3K gamma on the stimulation of GPCR [57]. Voltage-dependent calcium channel subunit alpha-2/delta-2 gene was found in this region, and PI3K could enhance native voltage-dependent calcium channel currents [58]. Diphosphoinositol polyphosphate phosphohydrolase 1 could cleave a beta-phosphate from the diphosphate groups in PP-InsP_5_. PP-InsP_5_ is similar to Ins (1,3,4,5) P_4_, the headgroup of PI (3,4,5) P_3_, which implied their competition relationship [59]. Chockalingam [60] reported that alpha 1-syntrophin could bind PI (4,5) P_2_. Hyaluronidase-2, a glycosylphosphatidylinositol-anchored receptor, could hydrolyze hyaluronic acid which could be degraded by *F. columnare* [2]. On linkage group 12, the genes within the suggestive QTL region seemed to be related with those within the QTL region on linkage group 7, because the paralogs of some genes in this region on linkage group 12 are found on linkage group 7 including phosphatidylinositol 3-kinase regulatory subunit 5 (*p101-PI3K*), phosphatidylinositol 3-kinase regulatory subunit 6 (*p87-PI3K*), guanine nucleotide-binding protein subunit beta 3, and voltage-dependent calcium channel gamma 6 subunit. On linkage group 14, spectrin beta chain, non-erythrocytic 2 *(βIII spectrin)* is located closest to the most significant SNP (AX-85234783). With a PH domain, βIII spectrin can bind PIP_2_ and get involved in membrane skeleton [61,62]. As presented in Figure 5, clearly many of these genes mapped in the significant QTL region or the suggestive QTL regions are involved in the related gene pathways.

It is notable that genes involved in the PI3K pathway were located together in “hubs” that were significantly associated with disease resistance. Theoretically, genes that are located together could be readily expressed in a coordinated fashion. However, here we do not have any evidence to indicate that the genes mapped within the QTLs involved in PI3K pathway are coordinately expressed. Analysis of RNA-seq data [20] indicated that some candidate genes indeed exhibited differences in baseline expression or after bacterial infection with columnaris. For instance, hyaluronidase-2 was expressed at a relative higher level in resistant fish than in susceptible fish before infection, and it was induced more in susceptible fish than resistant fish after infection. Guanine nucleotide-binding protein subunit beta-1, another gene that mapped within the suggestive QTL region, is expressed significantly higher in the susceptible fish than in the resistant fish. After infection, its expression in susceptible fish, but not in resistant fish, was drastically induced. However, because the experimental system is quite different, such expression data may not be directly transferable to our results here.

The most striking finding of our study was that the genes closest to the most significant SNPs were both positionally and functionally related, i.e., they are structurally organized as “functional hubs”. Although it is unknown at present whether these genes are expressed in a coordinated fashion, it was reported that neighboring genes tend to have similar expression patterns and get involved in related functions [63-65]. For instance, Schmid et al. [66] elucidated that genes in close proximity are much more likely to be co-expressed than expected by chance. Future analysis for coordinated expression of genes involved in PI3K pathway is warranted.

The QTLs identified in this study explained a limited fraction of the phenotypic variance of columnaris disease resistance. Firstly, the population specificity of QTLs is the most important reason why our family-based association mapping cannot detect all the QTLs associated with columnaris resistance [67]. Even within the same strain, various families showed drastically different susceptibilities to columnaris disease [68]. Secondly, segregating alleles within one species may lead to decreased power of analysis, especially in the case that one parental species systematically carries resistance alleles while the other one carries susceptibility alleles [69]. Thus the region cannot be detected with strong significance using intraspecific SNPs. The associated SNPs on linkage group 14 are intraspecific, so the effect of the locus may be underestimated and we cannot infer the origins of the resistant alleles. Thirdly, because of the lack of recombination between nearby QTLs, the locus with minor effect cannot be detected if the favorite allele on a close QTL with a major effect have a different origin. Lastly, but not leastly, genome level variations such as allele variations can account for only a fraction of phenotypic variations. Gene expression regulations at various levels such as transcriptional and posttranscriptional levels, as well as environment and genotype-environment interactions can have a profound impact on the final phenotype in performance.

## Conclusions

In summary, our GWAS using backcross interspecific hybrid population allowed mapping of associated QTLs and estimation of their effects for columnaris resistance. On linkage groups 7 and 12, the resistant genotypes were homozygous with both alleles from channel catfish. Examination of genes in the mapped QTL regions allowed further analysis of candidate disease resistance genes. It appears that signal transduction pathways involving PI3Ks may play a crucial role for disease resistance against columnaris. This notion is not only supported by the presence of PI3K pathway genes in the significantly associated QTL on linkage group 7, but also by the fact that many genes within the suggestive QTL on linkage group 12 are paralogs of those found on linkage group 7. In addition, many genes found within suggestive QTLs on linkage groups 7, 12 and 14 are also involved in PI3K pathway. Future studies are required for the identification of the causative genes for disease resistance. For example, GWAS using larger or more families can be conducted to increase the power and resolution. Ultimately, gene knockout experiments are needed to demonstrate the candidate genes as the disease resistance genes.

The most interesting discovery of this work is that functionally related genes that may be responsible for columnaris disease resistance are located closely in a limited number of positions, forming “functional hubs”. Future analysis of expression of genes in the PI3K pathway in relation to the resistance phenotype should determine whether the co-localized and functionally related genes are indeed expressed in a coordinated fashion.

## Methods

### Ethics statement

All experiments involving the handling and treatment of fish were approved by the Institutional Animal Care and Use Committee (IACUC) at Auburn University. Tissue samples were collected after euthanasia. All animal procedures were carried out according to the Guide for the Care and Use of Laboratory Animals and the Animal Welfare Act in the United States.

### Experimental fish, bacteria challenge and sample collection

The study population was the Auburn University one year old catfish generated from back cross of male F1 hybrid catfish (female channel catfish × male blue catfish) with female channel catfish. The backcross progenies were produced by using the F1 as the male parent to avoid possible maternal effects in the early growth stage. The population consisted of six families. Since the offspring were mixed for culture, the genotypes of the samples could be used to assign the offspring into their families (see Table 1). Three reasons make us to choose the backcross family-based population as the study sample. First, the channel catfish × blue catfish interspecific system provides a useful research system for the understanding of columnaris resistance because they exhibit clear contrast in their phenotypes [4]. Segregation of genotypes in F2, along with the highly contrasted phenotypes, provides a good system for QTL analysis. Second, family-based association mapping is usually more powerful in detecting QTLs, since the lack of recombination between a QTL and linked marker increases the power of detection [70]. Third, family-based association mapping is preferred to detect rare markers related with QTLs [70].

1200 fish (average body weight 55.3 grams) were randomly obtained from Auburn University Fish Genetics Facility and acclimated for one week in the aerated flow-through water [240 × 60 × 45 cm (L × W × H)]. The average water temperature was 25°C. A total of 340 backcross progenies were selected from the extremes of the disease resistance distribution of the 1200 fish based on the selective genotyping method [24]. The first 169 fish that died of columnaris were continuously sampled as the susceptible group. When the fish lost balance, blood samples were collected. 171 fish that survived from the disease and showed no symptoms were selected randomly as the resistant group.

The bacteria challenge procedure was conducted as previously described [18]. The bacteria *F. columnare* were provided by the Aquatic Microbiology Laboratory, Auburn University. To get a single *F. columnare* colony, several fish were experimentally infected with a virulent isolate (BGFS-27; genomovar II) [71], and bacteria were re-isolated from one symptomatic fish after confirmed visually and biochemically. We selected BGFS-27 as representative of *F. columnare* for the present study, to which channel catfish was more resistant than blue catfish [4]. A single colony was cultured in modified Shieh broth for 24 h in a shaker incubator (100 rpm) at 28°C. The final concentration of the bacteria was determined using colony forming unit (CFU) per mL. Challenge experiments were then conducted by immersion exposure for 2 h at a final concentration of 3 × 10^6^ CFU/mL. Control fish were treated with identical procedures except that they were exposed to sterile modified Shieh broth.

### DNA isolation, genotyping, and quality control

DNA was isolated using standard protocols. Briefly, the blood samples were incubated at 55°C overnight and DNA was extracted twice with phenol and once with chloroform. DNA was precipitated by isopropanol and collected by brief centrifugation, washed twice with 70% ethanol, air-dried, and resuspended in reduced EDTA TE buffer (10 mM Tris–HCl, 0.1 mM EDTA, pH 8.0). DNA was quantified using spectroscopy by Nanodrop (Thermo Scientific). The integrity of DNA samples was checked by 1% agarose gel electrophoresis stained with ethidium bromide. DNA was diluted to 50 ng/uL and the quality of genomic DNA satisfied the requirement for the genotyping platform of SNP arrays.

We have developed a catfish 250 K SNP array using Affymetrix Axiom genotyping technology [25,72]. Genotyping using the catfish 250 K SNP array was performed at GeneSeek (Lincoln, Nebraska, USA). The informative SNPs in this GWAS were distributed across the catfish genome at an average interval of 3.6 Kb. No sample was excluded due to low quality or low call rate (<95%). 214,797 SNPs were kept after filtering out SNPs with an inheritance or genotyping error, a minor allele frequency (MAF) <5%, or a call rate < 95%.

### Statistical analysis

Statistical analysis was carried out using the SVS software package (SNP & Variation Suite, Version 8.0). Pairwise linkage disequilibrium for the backcross progeny population was calculated according to r^2^ value. LD pruning was conducted with a window size of 50 SNPs, a step of 5 SNPs, and r^2^ threshold of 0.5, resulting in 14,420 independent SNP markers. The population structure was assessed by principal component analysis with the independent SNP markers.

EMMAX (Efficient Mixed-Model Association eXpedited) analyses using all SNPs were conducted with the first two principal components and the fish body weight as covariates [73]. The model is as follows:

$$ \mathbf{Y} = \mathbf{X}\mathbf{b} + \mathbf{Z}\mathbf{a} + \mathbf{e} $$

where **Y** is the vector of phenotype; **b** is the vector of fixed effects including weight and first two principal components; **a** is the vector of random effects with covariance kinship matrix **G**; **e** is the vector of random residuals; **X** and **Z** are coefficient matrices.

The threshold P-value for genome-wide significance was calculated based on Bonferroni-correction with the estimated number of independent markers and LD blocks [74]. A Manhattan plot of the P-value results was produced using the SVS software. Although channel catfish and blue catfish are two species, apparently their genome architecture is extremely similar according to our former studies and unpublished data [75-77]. Thus, the genetic marker map was constructed according to channel catfish genome sequence.

### Sequence analysis

The upstream and downstream genes of the significant SNPs were determined. GENSCAN program [78] and FGENESH+ [79] were used to analyze the catfish genome sequences (unpublished data) that surround the SNPs to identify the upstream and downstream genes. The identified genes were annotated by searching against the non-redundant protein database [80]. Genomicus [81] was utilized to construct the synteny of the counterpart genes from zebrafish to provide evidence for orthology.

## Availability of supporting data and requirements

No new SNPs were discovered in this manuscript. The SNPs used in this manuscript are from the catfish 250 K SNP array [25]. The data sets supporting the results of this article are included within the article. The other information can be obtained from the corresponding author upon request.
